# A comparative analysis of non-communicable disease risk among adolescents aged 12 to 18 in the Eastern Cape, South Africa, with regard to sex, school quintiles, and perceived stress

**DOI:** 10.3389/fpubh.2025.1672715

**Published:** 2025-11-20

**Authors:** Avela Mathe, Maria Maya van Gent, Rudolph Leon van Niekerk

**Affiliations:** 1 Department of Human Movement Science, University of Fort Hare, Alice, South Africa; 2 Department of Psychology, Social Work and Criminology, University of Fort Hare, East London, South Africa

**Keywords:** adolescents, metabolic risk factors, non-communicable diseases, perceived stress, South Africa

## Abstract

**Background:**

Non-communicable diseases (NCDs) are a growing concern in low- and middle-income countries, especially among adolescents. Recent data indicate a rise in NCD cases within this group. Despite the increasing prevalence of NCD risk factors, limited research has explored the relationship between perceived stress and NCDs.

**Study aim:**

To compare adolescents aged 12 to 18 years who are at risk for NCDs with those who are not at risk, considering sex, school quintiles, and perceived stress.

**Settings:**

The study involved 266 adolescents selected through stratified random sampling from seven schools in two Eastern Cape districts.

**Methods:**

It assessed anthropometric and physiological measurements and perceived stress levels. Descriptive statistics were used to summarize data, while independent samples t-tests analyses were employed to compare groups. Logistic regression was utilized to predict probability.

**Results:**

Most physical and physiological assessments were normal, except for females’ elevated BMI (24.52 ± 6.11), classifying them as overweight. Perceived stress did not differ significantly between at-risk and not-at-risk adolescents, although at-risk females reported higher stress levels than males. Perceived stress was consistently higher among adolescents from Quintiles 4–5 schools across both at-risk and not-at-risk groups. Logistic regression analysis indicated that sex was the significant predictor of NCD risk, with females being four times more likely to develop NCD risk factors than males, whereas school quintile was not a significant predictor.

**Conclusion:**

The study highlights a greater risk of NCD development among female adolescents, who also experience elevated levels of perceived stress. Recommendations to address these findings include specific strategies that reduce this population’s risk factors.

## Introduction

Non-communicable diseases (NCDs) disproportionately impact low- and middle-income countries (LMICs), where over 75% of NCD-related deaths occur, and are projected to account for nearly half of the global disease burden in developing countries (1–3). The development of NCDs is influenced by multiple factors, including genetic predispositions, unhealthy behaviors, and environmental risks that accumulate over time, with significant long-term impacts on adolescents (4). According to the World Health Organization (4), NCDs accounted for approximately 11% of all deaths among South African children and adolescents.

In recent years, adolescents have shown a high occurrence of risk factors for NCDs, including obesity, hypertension, and diabetes (5, 6). These risk factors encompass elevated blood glucose levels, excess body weight, increased cholesterol, and high blood pressure (7). The influence of these factors is further modulated by sex, body mass index (BMI), and socioeconomic status, which together contribute to the onset and severity of NCDs among children and adolescents (6, 8).

Overweight and obesity among children and adolescents are significant global health concerns (9). The 2019 South African NHANES found that 13.5% of rural South Africans aged 6 to 18 were overweight or obese, mainly due to poor nutrition and physical inactivity (4). Wrottesley (5) reported that between the ages of 15 and 19, 27% of females and 9% of males were overweight or obese, with higher rates in urban areas. Similarly, HAKSA (2018) reported that 16.1% of girls and 6.1% of boys aged 15–19 were overweight (4, 10). Debeila et al. (11) reported sex differences, noting that females are at a higher risk of developing obesity than males.

According to the International Diabetes Federation, 11.8% of South Africans have diabetes mellitus. While type 2 is more common, type 1 diabetes (T1DM) rises by 2–5% annually (12, 13). Type 1 diabetes (T1D) is one of the most common chronic conditions in adolescence, with over 1.1 million children under 20 affected globally. In South Africa, the prevalence among children aged 0–14 is approximately 0.8 per 100,000 and rising (14). Research on children and adolescents with type 1 diabetes is limited, particularly within the South African context.

Dookhony et al. (12) observed an incidence of dyslipidemia at 28.6% and an incidence of hypertension at 8.1%. Raised cholesterol is estimated to cause 2.6 million deaths annually, increasing the risks of heart disease and stroke (15, 16). High blood cholesterol is most common during adolescence, especially in adolescents over 16. However, being overweight or obese was not found to be related to raised cholesterol (12). Ozsu et al. (17) reported higher cholesterol levels among rural South African males aged 2–19 compared to females, with approximately 22% of males in school settings showing elevated cholesterol levels versus 15% of females. A study conducted among 27,358 children, adolescents, and young adults in Cape Town Dookhony et al. (12) observed an incidence of dyslipidemia at 28.6%. Additionally, high cholesterol affects 17% of children and adolescents aged 2–19, particularly those aged 16 and older (15–17).

Masocha et al. (18) found that 44% of boys and 46% of girls aged 15 and older were diagnosed with hypertension, with the highest occurrence among boys from lower socioeconomic backgrounds without formal education (18, 19). Studies from 2019 reported hypertension rates in South African children and adolescents ranging from 1 to 25.9%, with boys in rural Eastern Cape showing higher rates (21.62%) than girls (14.29%) (20, 21). Hypertension increased with age in rural adolescents (22), and boys in KwaZulu-Natal were more likely to be hypertensive than girls (23). Kamkuemah et al. (24) found that urban adolescents had higher mean diastolic values than those in rural areas. Letswalo et al. (25) also reported that elevated blood pressure in adolescents is linked to age, weight, height, and BMI. Obesity, overweight, and stress are believed to contribute to gender differences in adolescent high blood pressure, alongside lifestyle factors such as physical activity and sedentary behavior (26).

Importantly, physiological risk factors are not the only contributors to NCDs; high perceived stress, including mental disorders and depression, also play a significant role (27). Akseer et al. (28) noted that NCDs, including prevalent mental health issues such as depression and anxiety, constitute a significant concern for adolescents globally. Mental health challenges brought on by stress have a detrimental impact on adolescents’ daily routines and can lead to serious complications for their health and development into adulthood (29, 30). The socioeconomic difficulties faced by many adolescents in South Africa render them more vulnerable to stress, significantly affecting their mental health and predisposing them to NCD development (31, 32).

Although physiological risk factors and perceived stress are recognized contributors to adolescent NCD risk, few studies have simultaneously examined how these factors interact with socioeconomic status, measured via school quintile, among adolescents in the Eastern Cape, South Africa’s poorest province. Understanding these interactions is critical, as adolescents in lower-resource settings may face unique stressors that influence both mental and physical health. To address this gap, the present study aimed to compare adolescents aged 12 to 18 years who are at risk for NCDs with those who are not, considering the influence of sex, school quintiles, and perceived stress.

## Materials and methods

### Study design

This study formed part of a larger research project titled: “An intervention to address the physical, physiological, and psychological risk factors linked to non-communicable diseases among adolescents in the Eastern Cape, South Africa.” The study utilized a quantitative approach and a cross-sectional design.

### Setting

The research comprised learners aged 12 to 18 years from 7 selected schools. The research was conducted in two districts within the Eastern Cape Province, focusing on both rural and urban settings. It is the second largest in South Africa by land area, ranking it the third-most populous province. Within these two districts, four schools were randomly selected from each using a computer-based randomization tool. A dropout rate of 4% was observed, primarily due to one school withdrawing during the study.

### Inclusion and exclusion criteria

The study comprised adolescents aged 12 to 18, from grades 8 to 10, and included both male and female learners. Ethnic backgrounds and socioeconomic statuses were not specifically targeted, although the research was conducted in two municipalities. Pregnant female learners were not eligible for participation in the study.

### Study population and sampling strategy

**Figure 1:**
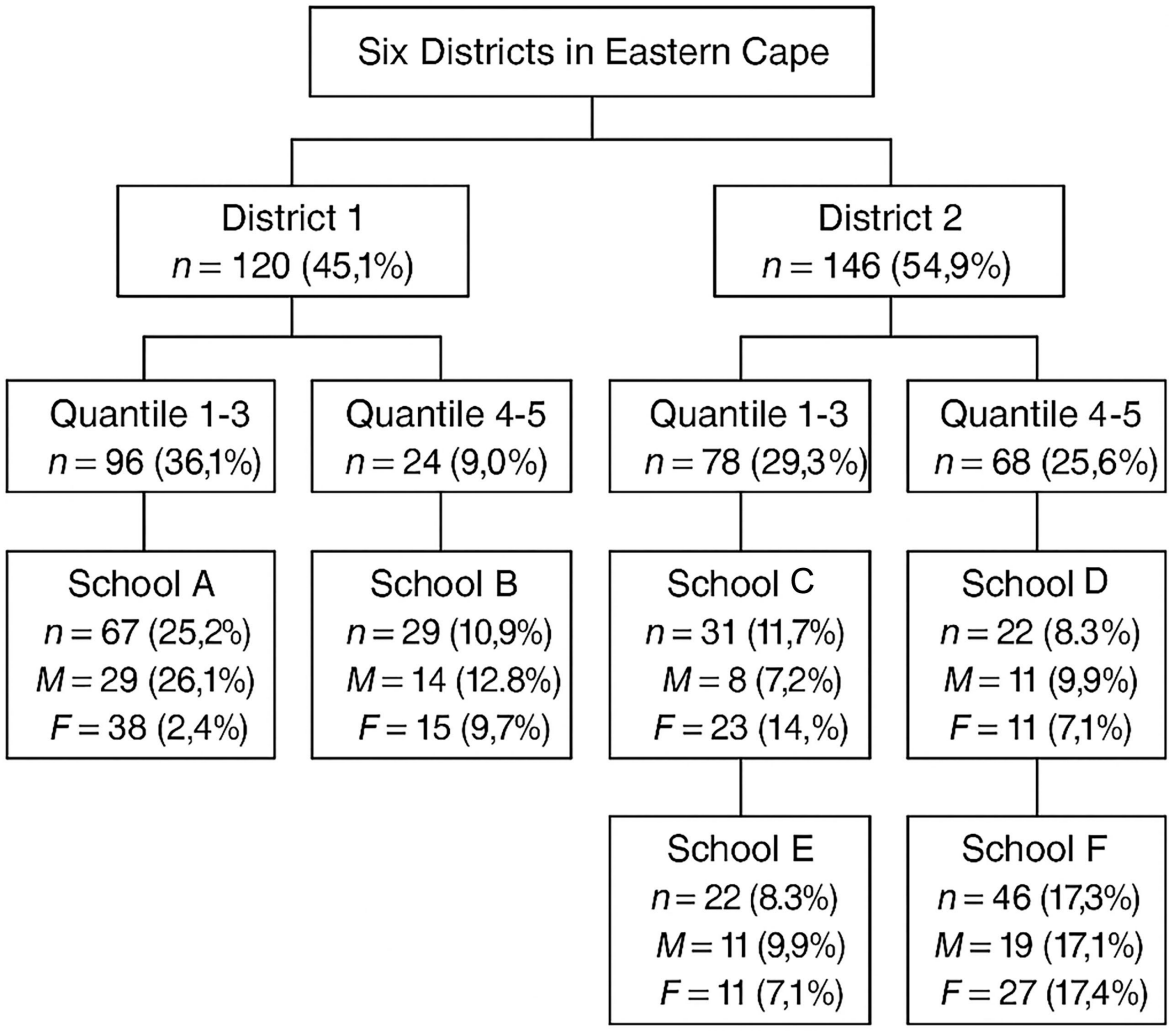
Illustration of stratified random sampling.

The total population of South African adolescents is approximately 9,950,100 (19), of which 2,473,140 are in the Eastern Cape. The final sample size was 266 adolescents, consisting of 111 (41.7%) males and 155 (58.3%) females. The sample of schools from quintiles one, two, and three (no fee-paying schools) represented socioeconomically disadvantaged groups, commonly referred to as the ‘poorest’ quintiles, whereas quintiles four and five schools (fee-paying schools) represented more affluent economic schools, referred to as the ‘least poor’ segment (33). Figure 1 illustrates the stratified random sampling methodology employed in this study.

### Data collection

As part of data collection, physiological risk factors for NCDs included blood pressure, blood cholesterol, random blood glucose, and body mass index. Adolescents are considered at risk for elevated blood pressure if their readings are between 120–129/80 mmHg, with hypertension defined as 130/80 mmHg or higher (4, 34, 35). Random blood glucose levels of 11.1 mmol/L or higher indicate that adolescents are at risk for diabetes (36, 37). Furthermore, HDL cholesterol levels above 4.4 mmol/L indicates a risk in adolescents. Adolescents’ BMI values were classified as follows: underweight (less than 18.5 kg/m^2^), normal weight (18.5–24.9 kg/m^2^), overweight (25.0–29.9 kg/m^2^), and obese (30.0 kg/m^2^ or higher) (10, 38). Additionally, the Perceived Stress Scale scores were interpreted within the following standard ranges: low stress (0–13), moderate stress (14–26), and high stress (27–40). The PSS demonstrated an internal consistency of Cronbach’s *α* = 0.632, which is acceptable for exploratory research, indicates modest reliability.

### Data analysis

Adolescents were classified as at risk if they showed one or more physiological risk factors: overweight/obesity, high systolic or diastolic blood pressure, elevated random blood glucose, or high blood cholesterol. Data analysis in this study utilized IBM SPSS software (version 2021) (55). IBM SPSS Statistics for Windows, Version 27.0. Armonk, NY: IBM Corp. A significance level of *p* < 0.05 was adopted, and confidence intervals were set at 95%. Descriptive statistics were presented as means and standard deviations. T-test Comparisons were made between male and female adolescents within the at-risk and not-at-risk groups for NCDs. Further comparisons were conducted between adolescents at risk and not at risk for NCDs across different school quintiles. Logistic regression was utilized to predict the probability of developing NCDs. The reliability of the Perceived Stress Scale was determined using Cronbach’s Alpha as a measure of the internal consistency of the instrument.

### Ethical consideration

This study was approved by the University Health Research Ethics Committee (Ref#2022 = 02 = 09 = AM). The Eastern Cape Department of Basic Education and school principals have granted consent for this research study. This was facilitated by presenting learners with a study information sheet and consent forms before data collection. The learners were requested to ask their parents or legal guardians for written informed consent to participate in the study. Additionally, all learners signed an assent form before participating in the study.

## Results

Table 1 compares male and female adolescents, with the mean values and standard deviations for anthropometric, physiological, and perceived stress measures. A significant difference in BMI was observed between males and females (*p* = 0.001), with females displaying higher mean BMI scores (24.52 ± 6.11) compared to males (20.18 ± 2.89), the mean value classifying female adolescents as overweight. Although not classified as at risk, males showed a slightly higher mean waist-to-hip ratio (0.77 ± 0.03) than females (0.73 ± 0.05). A significant gender group difference in perceived stress was found (*p* = 0.024), with females reporting slightly higher stress levels (19.70 ± 6.0) than males (18.10 ± 5.18), both reflecting moderate stress levels. None of the metabolic indicators exceeded clinical thresholds, suggesting that while group differences exist, the average values remain within expected healthy ranges.

**Table 1:** Comparison between 12 and 18-year-old male and female adolescents (Mean ± STD).

Variables	Males (*n* = 111)	Females (*n* = 155)	*p*-value	Cronbach’s alpha
Age (years)	15.34 ± 1.30	14.75 ± 1.13	0.001*	
Weight (kg)	55.61 ± 9.26	61.19 ± 16.33	0.001*	
Height (cm)	165.8 ± 7.29	157.81 ± 6.61	0.001*	
BMI (kg/m^2^)	20.18 ± 2.89	24.52 ± 6.11	0.001*	
Waist (cm)	67.94 ± 6.51	71.67 ± 11.22	0.002*	
Hip (cm)	88.31 ± 7.29	98.12 ± 12.19	0.001*	
Waist to hip ratio (n)	0.77 ± 0.03	0.73 ± 0.05	0.001*	
Systolic blood pressure (mmHg)	119.40 ± 14.57	117.97 ± 13.06	0.405	
Diastolic blood pressure (mmHg)	66.68 ± 13.71	68.31 ± 11. 91	0.304	
Random blood glucose (mmol/L)	4.02 ± 1.40	4.24 ± 1.40	0.213	
Blood cholesterol (mmol/L)	2.97 ± 1.54	3.53 ± 1.63	0.006	
Perceived stress (n)	18.10 ± 5.18	19.70 ± 6.01	0.024*	0.632

*Significance = *p* < 0.05; BMI, Body Mass Index.

For the sample of participants within the at risk group (*n* = 131; 50.4%) the prevalence of the risk factors are presented in Table 2. The highest prevalence of risk factors was observed among females (71%) and participants from less affluent schools (64.9%). More than half of the females (57%) and participants from more affluent schools (58.7%) presented with overweight and obesity. The highest prevalence for high blood pressure (31.6%) and elevated glucose (26.3%) was observed among males. High levels of cholesterol were observed among females (50.5%) and less affluent schools (51.8%).

**Table 2:** The prevalence of each individual risk factor within the at-risk group, broken down by sex and school quintile.

Variable	Total at risk (n)	Gender		School quintiles	
		Males *n* (%)	Females *n* (%)	Quintiles 1–3 *n* (%)	Quintiles 4–5 *n* (%)
At risk group	131	38 (29%)	93 (71%)	85 (64.9%)	46 (35.1%)
Overweight/obesity	61	8 (21.1%)	53 (57%)	34 (40%)	27 (58.7%)
High blood pressure	33	12 (31.6%)	21 (22.6%)	20 (23.5%)	13 (28.3%)
Elevated glucose	27	10 (26.3%)	17 (18.3%)	21 (24.7%)	6 (13%)
High cholesterol	63	16 (42.1%)	47 (50.5%)	44 (51.8%)	19 (41.3%)

Table 3 compares males and females within the at-risk group and the not-at-risk group in relation to metabolic risk factors and perceived stress variables. Female adolescents consistently showed significantly higher BMI and perceived stress levels (*p* < 0.05) than males, especially in the at-risk group. The not-at-risk males reported slightly higher stress than at-risk males.

**Table 3:** Comparison between 12–18-year-old male and female adolescents within at-risk and not at-risk groups for NCDs.

Variables	Adolescents not at risk (*n* = 131)			Adolescents at risk (*n* = 126)		
	Male	Female	*p*-value	Male	Female	*p*-value
BMI	19.36 ± 1.84	20.33 ± 2.12	0.007*	21.94 ± 3.86	26.75 ± 6.31	0.000*
Systolic blood pressure	117.10 ± 12.75	113.55 ± 10.67	0.102	123.50 ± 17.38	120.15 ± 13.83	0.260
Diastolic blood pressure	63.14 ± 11.16	66.09 ± 10.66	0.137	73.35 ± 16.28	69.43 ± 12.44	0.149
Blood glucose	3.90 ± 1.44	4.25 ± 1.03	0.133	4.25 ± 1.32	4.26 ± 1.57	0.955
Blood cholesterol	2.68 ± 1.52	2.95 ± 1.49	0.318	3.52 ± 1.45	3.83 ± 1.63	0.330
Perceived stress	18.57 ± 4.87	19.39 ± 5.97	0.398	17.08 ± 5.87	19.91 ± 6.18	0.022*

*Significance = *p* < 0.05; BMI, Body Mass Index.

In Table 4, statistically significant differences were observed between school quintiles for the at-risk group in terms of BMI (*p* = 0.000), with adolescents in quintiles 4–5 showing a higher mean BMI (27.91 ± 7.32) compared to those in quintiles 1–3 (24.00 ± 4.75). The only significant difference observed between quintile in the not-at-risk group was in terms of blood cholesterol (*p* = 0.002). According to the results, the other variable that presented a statistically significant difference between quantiles in both groups was for perceived stress (*p* = 0.001; *p* = 0.000), respectively, with quintile 4–5 (affluent schools) presenting higher values than quintile1-3 (disadvantaged schools).

**Table 4:** Comparison between 12–18-year-old adolescents at risk and not at risk for NCDs, in terms of school quintiles.

Variables	Adolescents not at risk (*n* = 131)			Adolescents at risk (*n* = 126)		
	Quintile 1–3	Quintile 4–5	*p*-value	Quintile 1–3	Quintile 4–5	*p*-value
BMI	19.75 ± 2.06	19.80 ± 1.94	0.896	24.00 ± 4.75	27.91 ± 7.32	0.000*
Systolic blood pressure	116.47 ± 12.39	113.80 ± 11.08	0.245	121.01 ± 15.78	121.04 ± 13.31	0.992
Diastolic blood pressure	65.67 ± 10.68	61.71 ± 11.33	0.058	71.28 ± 14.13	69.10 ± 12.68	0.374
Blood glucose	3.96 ± 1.33	4.22 ± 1.20	0.307	4.20 ± 1.55	4.36 ± 1.46	0.565
Blood cholesterol	3.08 ± 1.42	2.19 ± 1.52	0.002*	3.91 ± 1.46	3.49 ± 1.76	0.143
Perceived stress	17.8 ± 4.89	21.12 ± 5.65	0.001*	17.48 ± 5.14	21.94 ± 6.83	0.000*

*Significance = *p* < 0.05; BMI, body mass index.

Table 5 presents the results of a logistic regression analysis conducted to assess the impact of sex, school quintiles, and perceived stress on the likelihood of adolescents developing risk factors for NCDs. The overall model was statistically significant, χ^2^(3, N = 257) = 29.13, *p* < 0.005, indicating that it could reliably distinguish between adolescents at risk and those not at risk of developing NCDs. The model explained between 10.7 and 14.3% of the variance in NCD risk and correctly classified 66.1% of cases. Although this suggests that the model provides meaningful insights, it also indicates that other influential factors not included in the analysis likely contribute to NCD risk for example diet, physical activity and genetics. Of the three independent variables, only sex contributed significantly to the model (*p* = 0.000). Female adolescents were found to be four times more likely to develop NCD risk factors compared to their male counterparts.

**Table 5:** Logistic regression analysis of the effects of sex, school quintiles, and stress on NCD risk among 12–18-year-old adolescents.

Variables in the equation	B	S.E.	Wald	Df	Sig.	Exp(B)	95% CI EXP(B)	for
							Lower	Upper
Sex	1.408	0.274	26.361	1	0.000*	4.088	2.388	6.998
School quintile	0.351	0.294	1.427	1	0.232	1.421	0.798	2.528
Stress	−0.018	0.024	0.565	1	0.452	0.982	0.936	1.030
Constant	−0.561	0.466	1.451	1	0.228	0.570		

Variable(s) entered on step 1: Sex, School quintiles Stress.

*Significance = *p* < 0.05.

## Discussion

This study aims to compare adolescents aged 12–18 who are at risk and not at risk for NCDs, considering sex, school quintiles, and perceived stress, and to examine how these factors influence the likelihood of NCD risk.

The results showed that female participants have a higher mean BMI score, indicating that, on average, female adolescents were classified as overweight, compared to their male counterparts. Consistent with the findings of Otitoola et al. (36), who conducted their study in Cofimvaba, a rural area of the Eastern Cape, a greater proportion of female adolescents (33.3%) were classified as overweight compared to their male counterparts (15.7%). Similarly, Wrottesley et al. (5), in a study conducted in Soweto, Gauteng, found that by the ages of 15–19 years, 27% of females and 9% of males were overweight or obese. In support of this, Debeila et al. (11), in the study conducted in rural areas of the Limpopo Province, also reported that females are at a significantly higher risk of obesity than males. These results highlight a gendered trend in excess weight gain among South African adolescents, with females more likely to be overweight or obese than males. Differences may influence females’ consistently higher BMI scores in physical activity, dietary habits, and sociocultural or environmental factors.

The result showed that gender differences were evident in the distribution of NCD risk factors among adolescents, with females showing a higher overall prevalence of risk factors and elevated cholesterol levels, whereas males presented with higher levels of elevated blood pressure and glucose. These findings suggest distinct patterns of NCD risks between sexes, consistent with previous research among South African adolescents reporting higher blood pressure and glucose levels among boys, and greater cholesterol levels among girls (1, 39). Similarly, studies conducted in the North West Province and Western Cape have shown that adolescent males are more prone to elevated systolic blood pressure, while females display higher BMI and total cholesterol concentrations (37, 39). These differences may be attributed to biological and hormonal variations, as well as behavioral patterns such as diet and sedentary behavior that differ between sexes during adolescence. The study observed a socioeconomic inconsistency. Participants from less affluent schools demonstrated a higher overall prevalence of risk factors and elevated cholesterol, overweight and obesity were more prevalent among those attending more affluent schools. Similar trends have been reported in urban South African adolescents (40, 41), where adolescents from higher socioeconomic backgrounds are more likely to consume energy-dense foods and engage in sedentary behaviors.

The results revealed that males had a slightly higher waist-to-hip ratio compared to their female counterparts. Similarly, a systematic review on obesity prevalence in South African children and adolescents noted that WHR tends to differ by sex, with boys often showing higher central adiposity despite lower overall body fat percentages (38). Jones et al. (42), in a study conducted in rural areas of South Africa, found that male adolescents had significantly higher WHR values compared to their female Counterparts.

These sex differences in fat distribution may be linked to hormonal, behavioral, and developmental factors and highlight the importance of including WHR as a complementary measure to BMI in adolescent health assessments. Internationally, a study by Ibrahim et al. (43) conducted in Dhaka, Bangladesh, a low- and middle-income country LMIC also identified WHR as a strong predictor of health risk, with 27.5% of males and only 6% of females classified as at risk. These findings suggest that even when BMI falls within normal ranges, elevated WHR in males may still indicate a predisposition to central obesity and be associated with NCDs.

The results found that male and female adolescents experience moderate stress levels, although females showed higher mean perceived stress scores. Östberg et al. (44), in a study conducted among adolescents in Stockholm schools, Sweden, found similar results, reporting that girls experienced higher levels of perceived stress than boys. Furthermore, the findings are consistent with those from South African studies conducted in Soweto and Durban, which reported a higher perceived stress among adolescent females than males. Factors such as intimate partner violence, depression, anxiety, poor self-rated health, and unhealthy lifestyle behaviors were identified as key contributors (15). Additionally, all adolescents in both groups had moderate stress; however, males at risk presented higher perceived stress scores compared to males not at risk. With limited recent research both internationally and in South Africa, comparing available studies helps to contextualize findings and identify common sex differences in adolescent stress patterns.

The results showed the significant differences between school quintiles for the at-risk group in BMI, with quintile 4–5 (affluent schools) presenting higher BMI scores compared to quintile 1–3 (disadvantaged schools). This is consistent with findings from a South African study by Reddy et al. (45), which reported an overweight and obesity prevalence of 25.3% among adolescents attending higher-income schools, compared to 16.8% among those in lower-income schools. Similarly, Gradidge et al. (21) found that overweight prevalence among urban South African adolescents was 27% in girls and 9% in boys, with higher BMI values associated with more affluent schools.

Perceived stress was consistently higher among adolescents from (Quintiles 4–5) schools across both at-risk and not-at-risk groups. This finding suggests that socioeconomic advantage does not necessarily protect against psychological stress and may, in some cases, be associated with increased academic and social pressures. Adolescents from affluent schools often face high performance expectations, competitive environments, and parental pressure, contributing to chronic psychological strain despite greater access to resources (46, 47). However, this finding contrasts with other studies conducted in South Africa and similar low- and middle-income contexts. For instance, a study in Cape Town reported that adolescents from lower socioeconomic backgrounds (Quintiles 1–3) experienced higher levels of perceived stress, largely linked to financial hardship, exposure to violence, and limited access to psychosocial support (48). Comparable trends have been documented in Nairobi, Kenya (49), and Khayelitsha, South Africa (26), where socioeconomic disadvantage is strongly associated with elevated stress. These contrasting patterns indicate that the relationship between socioeconomic status and perceived stress varies by context, reflecting diverse sources and appraisals of stress. Moreover, perceived stress does not always align with metabolic risk patterns and may be shaped by broader psychosocial and environmental influences. Overall, these findings highlight the complexity of stress among South African adolescents and the need for context-specific strategies to address it across school quintiles.

The study demonstrated a significant difference between quintiles in the not-at-risk group regarding blood cholesterol, with quintile 1–3 (disadvantaged school) presenting higher scores than quintile 4–5 (affluent schools). This is consistent with findings from a South African study, where 21.8% of adolescents from low socioeconomic backgrounds had elevated total cholesterol, compared to 12.5% from higher-income groups (45). Similarly, in Brazil, 27.6% of adolescents in low-income public schools had high cholesterol levels, compared to 14.3% in wealthier, private school settings (50). Including data from Brazil, a country with similar socioeconomic inequalities, adds valuable context, mainly due to the limited research on adolescent cholesterol and socioeconomic status in South Africa.

Female adolescents were determined to be 4 times more likely to develop NCDs compared to their male counterparts. This finding highlights that sex is a strong predictor of NCD risk in adolescents. Due to the limited availability of sex-specific NCD data among adolescents in South Africa, data from comparable low- and middle-income countries (LMICs) were used for comparison. A global survey of adolescents from low- and middle-income countries reported a higher clustering of NCD risk factors among female adolescents (51). In Nepal, Uddin et al. (52) found that adolescent girls were significantly more likely than boys to exhibit unhealthy dietary behaviours and insufficient physical activity. Similarly, in Bangladesh, Urmy et al. (53, 54) and Hassan et al. (37) reported that adolescent girls had a higher prevalence of combined behavioral and physiological risk factors, including poor diet, physical inactivity, and lipid abnormalities. These findings suggest that female adolescents are more prone to developing NCDs, likely due to a combination of biological susceptibilities, gendered social norms, and limited access to health-promoting resources.

### Limitations

The first limitation of this study was the reduced sample size resulting from the withdrawal of one school from participation, which led to the failure to achieve the desired sample size. The second limitation pertains to the sample, which consists of adolescents from two municipalities in the Eastern Cape region. This specific population may not fully represent all adolescents residing in the Eastern Cape or South Africa. Consequently, the findings derived from this study may not be generalizable to the broader adolescent population in the region or country. A further limitation concerns the Perceived Stress Scale (PSS), although validated and widely used among adolescents, demonstrated modest internal consistency (*α* = 0.632) in this sample. This suggests that responses may have been influenced by contextual factors. Future studies should consider using culturally adapted or complementary stress measures to enhance reliability.

## Conclusion

This study aims to compare adolescents aged 12–18 who are at risk and not at risk for NCDs, considering sex, school quintiles, and perceived stress, and to examine how these factors influence the likelihood of NCD risk. The results showed that perceived stress and school quintile were insignificant predictors of NCD risk. However, sex, particularly being female, emerged as a significant predictor, with female adolescents demonstrating a higher likelihood of being at risk, as indicated by elevated BMI and related health markers. Although perceived stress was not directly associated with NCD risk, female adolescents reported higher stress levels compared to their male peers. Contrary to existing literature, adolescents from lower-income school quintiles reported lower levels of perceived stress than those from higher-income quintiles. These findings suggest that the relationship between socioeconomic status and stress may be more complex in this context and warrants further investigation.

These findings highlight the need for sex-sensitive public health strategies targeting adolescent females and suggest that biological and behavioral factors may exert a more substantial influence on early NCD risk than socioeconomic or psychosocial variables. Furthermore, the limited availability of high-quality, context-specific research on adolescents in low-quintile South African schools highlights the need for more robust investigations. Future studies should explore school-based interventions and adopt multilevel designs that consider demographic, environmental, and societal influences to fully capture the complexity of NCD risk development in adolescents.

## Data Availability

The original contributions presented in the study are included in the article/supplementary material, further inquiries can be directed to the corresponding author.
